# Description of Poly(aryl-ether-ketone) Materials (PAEKs), Polyetheretherketone (PEEK) and Polyetherketoneketone (PEKK) for Application as a Dental Material: A Materials Science Review

**DOI:** 10.3390/polym15092170

**Published:** 2023-05-02

**Authors:** Syazwani Mohamad Zol, Muhammad Syafiq Alauddin, Zulfahmi Said, Mohd Ifwat Mohd Ghazali, Lee Hao-Ern, Durratul Aqwa Mohd Farid, Nur A’fifah Husna Zahari, Aws Hashim Ali Al-Khadim, Azrul Hafiz Abdul Aziz

**Affiliations:** 1 Faculty of Dentistry, Universiti Sains Islam Malaysia (USIM), Kuala Lumpur 55100, Malaysia; 2 Department of Conservative Dentistry and Prosthodontics, Faculty of Dentistry, Universiti Sains Islam Malaysia (USIM), Kuala Lumpur 55100, Malaysia; 3 Department of Basic Sciences and Oral Biology, Faculty of Dentistry, Universiti Sains Islam Malaysia (USIM), Kuala Lumpur 55100, Malaysia; 4 SMART RG, Faculty of Science and Technology (FST), Universiti Sains Islam Malaysia (USIM), Nilai 71800, Malaysia; 5 Department of Paediatrics Dentistry and Orthodontics, Faculty of Dentistry, Universiti Sains Islam Malaysia (USIM), Kuala Lumpur 55100, Malaysia

**Keywords:** additive manufacturing, dental materials, dentistry, high-performance polymer (HPP), polyetheretherketone (PEEK), polyetherketoneketone (PEKK)

## Abstract

Poly(aryl-ether-ketone) materials (PAEKs), a class of high-performance polymers comprised of polyetheretherketone (PEEK) and polyetherketoneketone (PEKK), have attracted interest in standard dental procedures due to their inherent characteristics in terms of mechanical and biological properties. Polyetheretherketone (PEEK) is a restorative dental material widely used for prosthetic frameworks due to its superior physical, mechanical, aesthetic, and handling features. Meanwhile, polyetherketoneketone (PEKK) is a semi-crystalline thermoplastic embraced in the additive manufacturing market. In the present review study, a new way to fabricate high-performance polymers, particularly PEEK and PEKK, is demonstrated using additive manufacturing digital dental technology, or 3-dimensional (3D) printing. The focus in this literature review will encompass an investigation of the chemical, mechanical, and biological properties of HPPs, particularly PEEK and PEKK, along with their application particularly in dentistry. High-performance polymers have gained popularity in denture prosthesis in advance dentistry due to their flexibility in terms of manufacturing and the growing interest in utilizing additive manufacturing in denture fabrication. Further, this review also explores the literature regarding the properties of high-performance polymers (HPP) compared to previous reported polymers in terms of the dental material along with the current advancement of the digital designing and manufacturing.

## 1. Introduction

**Figure 1:**
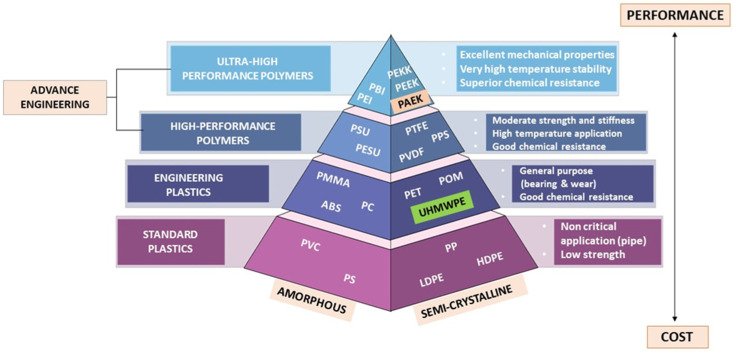
Polymer pyramid listing high performance polymers used as dental materials organized from high-performance polymers plastics to low cost in term of amorphous and semi-crystalline.

A polymer can be defined as a substance which has a molecular structure built up from a large number of similar units (called monomers) bonded together. The simplification type of polymer is depicted as in Figure 1, which identifies three major categories of polymers in dental material, i.e., standard plastics polymer, engineering plastics polymer, and advance engineering polymer.

According to the polymer pyramid, high-performance polymers (HPPs) belong to the uppermost class of plastics, being much more difficult to produce, based on more complex monomers, and generally more expensive as a result of possessing better temperature and chemical stability and mechanical properties than the commodity plastics, but typically being manufactured in lower volumes. Polyaryletherketone (PAEK) is a crystalline polymer formed by linking phenylene rings through oxygen bridges and carbonyl groups (ketones). According to different structures, PAEK mainly includes polyetherketone (PEK), polyetheretherketoneketone (PEEKK), polyetherketoneetherketoneketone (PEKEKK), polyetherketoneketone (PEKK), and polyetheretherketone (PEEK) [1].

In the past 20th century, denture bases made of plastic-like materials were increasingly popular since they were less expensive. A removable partial denture (RPD) comprises three main components, namely resin for the base, replacement teeth, and metal for the structure. Most denture base resin comprise of the polymethyl methacrylate (PMMA), whilst the type of prosthetic teeth will depend on the clinical scenario [2]. Poly (methyl methacrylate) (PMMA) was established in practical use as acrylic resin for the fabrication of dentures in 1936 [3]. Notwithstanding, the first PMMA utilized in denture bases was a heat-processed derivative. Soon after, the researchers discovered a new technique called auto-polymerizing acrylic resin, which can fully cure without heat. However, the auto-cured acrylic resin hardens immediately after blending, significantly limiting the time used for manipulation. In response, scientists created light-cured acrylic resin, which must be used with a device that uses light to initiate the curing reaction, such as an argon-ion laser or a filtered halogen lamp emitting blue light [3].

Despite the advances in preventive dentistry, removable denture (complete and partial) treatments are non-invasive methods to relieve geriatric patients’ physical and emotional strain, rehabilitate oral function, and enhance their ability to adjust psychologically and socially [4]. The World Health Organization (WHO) deemed the loss of natural teeth and underlying alveolar bone impairment in 1980 since it involved losing an anatomical body part [5]. Partial dentition and edentulous are rising because of the longer life expectancy along with an increase in the population’s elderly citizens. Along with growing older and losing teeth, the bones and soft tissues deteriorate over time. Nonetheless, dental caries is a persistent condition [6], which is also the main reason for tooth loss and mouth pain. Hence, a denture is necessary as an effort to address the issue of tooth loss.

Metal was the primary component of the early dentures, which were made from it in their entirety. On the other hand, there are a few drawbacks, such as the possibility of hypersensitivity, metal display that causes aesthetic problems, oral galvanic reactions, unfavorable tissue reactions, abutment teeth osteolysis, and the production of biofilm [7,8]. Due to the limits of metal-based frameworks, research has been done on non-metal materials, particularly polymers, such as polyethylene glycol, polymethyl methacrylate (PMMA), and aryl-ketone polymers [8]. Polymers are typically viscoelastic, insulative materials having a wide range of industrial, aerospace, and biomedical applications. Based on previous research, computational evaluation of the contact pressure on a metallic bearing of a hip joint prosthesis is mostly done for metal-on-metal, and it is still rare for research focusing on metal-on-UHMWPE to investigate the choice of metal material to reduce contact pressure that is useful as a preliminary study before evaluating wear [9].

However, the disadvantages of polymer frameworks include the fact that they are more difficult to masticate than metal, have mechanical characteristics similar to those of metal, and have a high coefficient of thermal expansion. The frameworks also have a low elasticity modulus, degrade more quickly than metal, and could be cytotoxic due to chemical leaching. A new group of such materials have been developed in recent years from the generic group of polyaryletherketone (PAEK). Within this group, the material polyetheretherketone (PEEK) and polyetherketoneketone (PEKK), a semicrystallize thermosoftening polymer, has the potential for a framework for fixed and removable prostheses by virtue of its biocompatibility and desirable physical and mechanical properties.

With the advancement of technology, new injectable polymer materials; high-performance polymer (HPP) have been developed for removable and complete dentures as a one type of transpire materials in dentistry. The description of Poly(aryl-ether-ketone) materials (PAEKs), namely polyetheretherketone (PEEK) and polyetherketoneketone (PEKK), for application as a dental material was highlighted in the present literature review, providing an insight into the newly developed HPP materials used as oral prosthetics, particularly denture bases, and allowing for a comparison with the commonly used polymethylmethacrylate (PMMA). They may help patients who want metal-free dental restorations because of their biocompatibility and sturdy mechanical precise mechanism. These properties include resistance to heat, solvent, fatigue, excellent electrical insulation, and robust wear [10,11]. There is a significant amount of current research and intent interest in HPPs as substitute materials with mechanical qualities comparable to human bone. Because of these qualities, HPPs are an attractive alternative to ceramic and metal as a material for restoration.

This review was conducted in PubMed, Google Scholar, and Ovid using as keywords additive manufacturing, high-performance polymer, PEEK, and PEKK with different dental disciplines, such as high-performance polymers in dentistry, prosthodontics, and dental material. This study intends to outline the benefits of HPP, notably PEKK and PEEK, to enhance the intraoral biomechanical and tribological response to dentistry application. Nonetheless, limited literature has highlighted the properties and description of poly(aryl-ether-ketone) materials (PAEKs), polyetheretherketone (PEEK) and polyetherketoneketone (PEKK), as a dental material, particularly in dentures introduced to alleviate the qualities of the denture base and reduce an array of issues affecting the denture base that might affect denture wearers. Previous research has mostly considered dental applications and there is a scarcity of studies focusing on the properties of high-performance polymers, particularly PAEKs.

## 2. Properties of High-Performance Polymers (HPPs)

**Figure 2:**
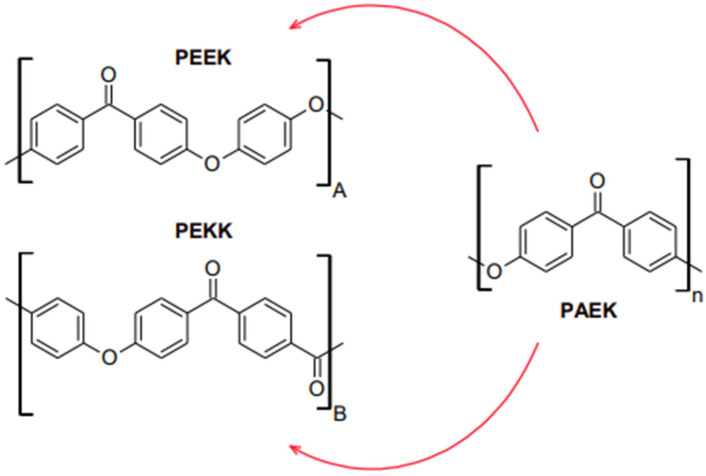
Reiterating unit construction of (A) Polyether ether ketone (PEEK), (B) Polyether ketone ketone (PEKK), from (n) Polyaryletherketone (PAEK).

According to the polymer pyramid in Figure 1, a high-performance polymer (HPP) is an advance engineering polymer that varies from ultra-high molecular weight polyethylene (UHMWPE) in terms of development, properties, and average molecular weight and chain length. High-performance polymers are located at the top of the polymer pyramid. The process of making HPP is much more difficult, based on the need for more complex monomers providing advantages, including low potential to deduce an allergy, low water solubility, superior biocompatibility, high thermal and chemical resistance, moderate biofilm formation, and excellent mechanical properties, which makes them more expensive [11,12]. HPPs are also made up of repeating units of macromolecules that give rise to long polymeric chain structures in three dimensions. Figure 2 shows the reiterating units of PEEK, PEKK, from PAEK that build up their respective polymer structures.

In contrast to restorations made of metal, HPPs are naturally radiolucent, making them easier to image using diagnostic imaging modalities such as X-ray, computed tomography, and magnetic resonance imaging [13]. In the field of dentistry, HPPs find use in various applications, including those on healing and transitional abutments [14]. Dental implants [15], clasps for dental implants, alternative inflexible materials for the frames of removable partial dentures [16], and fixed dental prostheses [10] are also examples of this type of innovation. The acronym HPP stands for high-performance polymer belonging to the polyaryletherketone (PAEKs) family of polymers. Thermo-pressing processes, such as those utilized by BioHPP^®^, Bredent, and Senden, can produce HPP devices. Alternatively, computer-aided design and manufacturing (CAD/CAM) techniques can mill HPP devices [17]. Consequently, HPPs are unsuitable for monolithic cosmetic dental restorations due to their low translucency and opaque coloration that can be described as either greyish or pearl-white. A veneer constructed of resin composite may be required to achieve an acceptable level of visual quality [10].

Polyaryletherketones (PAEKs) are an entire family of thermoplastics polymers that consist of aromatic rings joined by ether or ketone linkages, as shown in Figure 2. There are no branches in these highly aromatic polymers, which exemplifies the order of nature characteristic of PAEKs polymers and adds to their exceptional resistance to chemicals, radiation, heat, and other desired physical qualities typical of high-performance polymers. Despite their reputation as rigid polymers, PAEK exhibits some degree of flexibility due to alternating ketone and ether linkages in their structure. As mentioned, PEEK and PEKK are the two most significant chemical components of the polymer.

In 1990, polyetheretherketone (PEEK) was the first biomedically relevant high-performance polymer (HPP). It is a homopolymer having a single monomer [18]. The chemical composition and structure (branching of polymer) of PEEK render its stability that facilitates its processing at high temperatures [19]. It is used in engineering medical applications because of its excellent thermal properties, superior wear resistance, outstanding processability, inertness, corrosion resistance, high strength, and modulus of elasticity [7]. Soon after PEEK synthesis, it was increasingly used in orthopedic, traumatic surgery, and spine implants [20]. In dentistry, PEEK could be advocated for use in oral implantology. According to the study, PEEK implants have become an alternative for patients with bruxism or even allergies to metal [21]. PEEK was introduced in many applications due to its unique physical properties. It is extensively applied as a substitute for metal in various contexts due to its improved performance compared to typical polymeric resins. PEEK’s advantageous characteristics include excellent biocompatibility, high mechanical and thermal qualities, chemical resistance, white color, and low specific weight. PEEK’s high elasticity enabled it to function while minimizing stress and distal torque on the abutment teeth. The reinforced variant of PEEK has a similar Young modulus (18 GPa) to human cortical bone, making it an “isoelastic” implant material [22].

**Figure 3:**
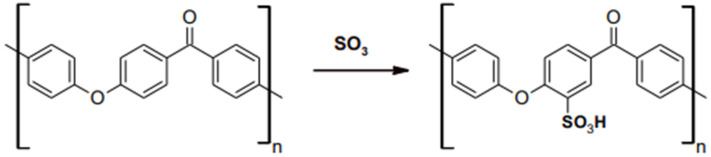
Sulfonation reaction on PAEK.

Other HPPs on the market comprise polyetherketoneketone (PEKK). Recently, the trend of utilizing PEKK in the medical and dental field has increased due to its desirable and promising biomechanical properties, such as compressive, tensile, and flexure strength. Apart from that, the addition of ketone groups within its molecular structure makes the material more versatile in surface modifications, bonding and improved melting temperature compared to PEEK [23]. The sulfonation reaction shown in Figure 3 present a -SO_3_H on the phenyl rings attached to ether and ketone group [24]. More ketone groups in PEKK increases the ability of surface chemical modification since the presence of -SO_3_H will be greater on PEKK than PEEK [23]. The elastic modulus of PEKK is also considerably similar to bone and has shock absorbance properties in a simulated intraoral environment [25]. Meanwhile, PEEK is an otherwise unclassified thermoplastic material. It can have a moderately high tensile strength among the thermoplastics in the database [26].

There is a significant association between the surface roughness and surface free energy of the processed surface and the ensuing color stability. Thermoplastic high-performance polymers (HPP) have sparked a resurgence in interest in dentistry. Nevertheless, the pressed and milled PEKK’s color is significant because of its remarkable properties. Food dyes like nicotine, anthocyanidins, tannins, and caffeine are examples of outside forces that can cause discoloration [27]. As described by A. Wildburger in his 2013 article “Colour Stability of Different Composite Resin Material”, diverse internal and external variables might cause discoloration of dental prosthesis. Internal factors include the type of resin matrix, percentage and filler size, composition and polymerization procedure, restorative material chemical interactions, age, and restoration processing mode.

**Figure 4:**
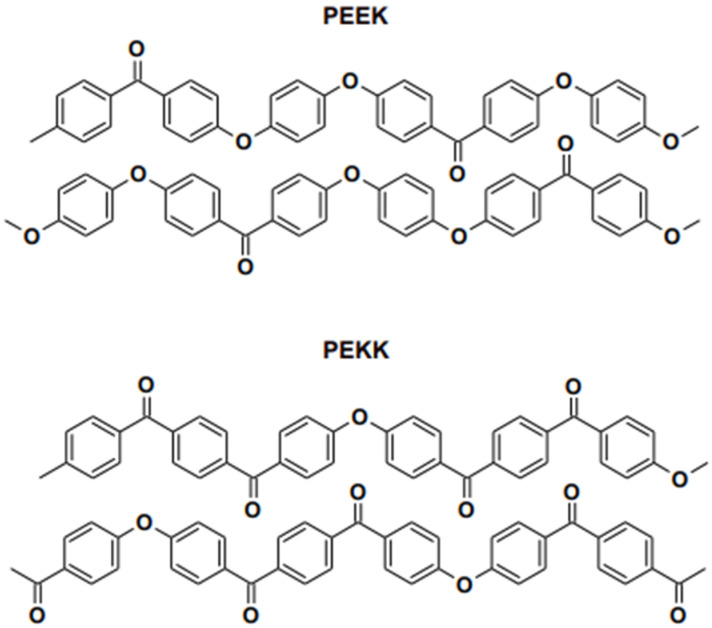
Chain packing in PEEK and PEKK as reported in literature.

These materials were invented to replace conventional metal alloys and ceramics to fabricate various fixed restorations and removable prostheses. Subjectively, PEEK materials are more appealing than metallic frameworks because of their greyish-brown or pearl-white opaque shade; metallic frameworks as fixed restoration frameworks necessitate veneering with composite resin [28]. Therefore, it is challenging to evaluate optical characteristics and color stability after artificial ageing and staining due to surface processing and finishing. The crystallinity of PEEK is higher than the crystal structure of PEKK [29]. PAEK polymers tend to crystallize as a consequence of chain packing induced by the ether and ketone linkages depicted in Figure 4. Compared to the ether linkages, the ketone linkages are less flexible and slow the packing of chains. Moreover, the crystallization. PEEK contains less ketone compared PEKK and the crystallinity of the material is higher.The focus of this literature review will encompass an investigation of the development of HPPs, emphasizing their application, particularly in dentistry, as material uses.

## 3. Properties of PEEK

PEEK’s outstanding qualities have made it “a wonder material” for use in various applications, including those in dentistry and medical. PEEK belongs to the ketone polymer subgroup. As illustrated in Figure 4, it is a semi-crystalline thermoplastic with a linear, highly aromatic molecular backbone that includes ether and ketone bonds. Because of their crystalline nature, PEEK resins have a great mix of physical qualities, including strength, chemical resistance, and hydrolytic thermal stability, due to their crystalline structure. PEEK is frequently utilized in the medical field, where it has been identified as a superior alternative to titanium in orthopedics [30].

Synthesis of PEEK involves the alkylation of bisphenol salt and aromatic nucleophilic substitution. The addition of hydrochinon salt to 4,4′-difluoro benzophenone caused a widespread response. Specific rigidity is provided by the presence of aromatic rings (benzene). The presence of an ether (-o-) bond demonstrates a second feature: the molecule is free to rotate about its axis in this orientation. When a molten molecule is gradually cooled, two different microstructure phases emerge. The material is stable at high temperatures (above 300 °C), resistant to chemical and oxidative deterioration, more powerful per mass, and compatible with reinforcing agents like glass and carbon fibers due to its chemical composition [31]. PEEK is unique since it can be supported with various materials, such as glass or carbon fibers. However, PEEK is applied in medical and dental applications due to its radiolucency, stability, biocompatibility, and mechanical properties since PEEK does not absorb radiation.

All of the excellent properties have elevated PEEK’s appeal in dentistry. Still, its hydrophobic and chemically inert surface limits its clinical uses, notably bonding with dental resin composites. The most general biomedical applications of PEEK materials include structural modifications and surface functionalization. Furthermore, due to its good biomechanical and stable chemical properties, PEEK is an intriguing synthetic polymer for use as an endoprosthetic material for ligamentous replacement. Hence, PEEK-based materials are rapidly emerging as a significant class of biomaterials used in various medical applications, including the replacement of bone and cartilage.

On the other hand, polyetheretherketone, also known as PEEK, is a polymer that is non-toxic and has an elastic modulus comparable to that of human bone. Compared to metal implants, PEEK implants provide several benefits, the most notable of which are good performance, a color analogous to teeth, and the absence of a stress-shielding effect. This material is less heavy than conventional ones, can adjust to a wide range of retentive forces, and can be easily retrieved through CAD/CAM manufacture. Incorporating these materials into the 3D printing process will help the technology find more widespread use.

## 4. Properties of PEKK

Polyetherketoneketone (PEKK) is a newly evolving polymeric material. The remarkable features of PEKK have captivated the interest of researchers due to its excellent properties in many applications, such as in oral implantology and prosthodontics [17]. Figure 3 depicts the structure of PEKK, which has a second ketone group that enhances the glass transition and melting temperature, increasing polarity and backbone rigidity [32]. In addition, the additional ketone group in PEKK possesses robust polymer chains and demonstrates improved physical and mechanical qualities, such as compressive strength [33]. Polyetherketoneketone (PEKK) is a helical semi-crystalline thermoplastic polymer comprising a series of ether or ketone groups bonded to a benzene ring, as depicted in the figure.

Furthermore, PEKK possesses both amorphous and crystalline behavior, allowing it to produce various products. Since PEKK resins are polymorphic, the crystal structure might vary from PEEK’s, depending on the conditions under which it is crystallized. Two different isomeric forms are included as repeating units along the polymer chain when the second ketone group is added to the polymer backbone of PEKK polymers. Recently, Polyetherketoneketone (PEKK) has been introduced in dentistry and has been used to make prosthetics and implants that work well. The remarkable biocompatibility of this material has led to its development as a potential long-term orthopedic replacement for titanium. A study by Stawarczyk B. et al. states that PEKK has an appropriate level of fracture resistance, and its ability to spread stress and absorb shock points to its potential for development as a new restorative material to replace metals and ceramics [34]. The fracture resistance, adequate strength (65 MPa), and stress absorption capabilities of PEKK boost its potential for use as a restorative material. Compared to dentin, the PEKK has a comparable compression strength but a lower modulus of elasticity.

In terms of chemistry, the second ketone group in PEKK makes it easier to change surface chemical modification than PEEK [35]. A novel strategy involves preparing bioactive surface-porous PEKK developed by a unique combination of the addition of HA microsphere porogen, with subsequent acid sulphonation treatment and biomimetic mineralization via simulated body fluid (SBF) incubation [36]. Although achieving some improved properties, a single physical or chemical treatment method in previous studies could struggle to improve the osteointegration property and bone ingrowth of porous PEEK scaffolds significantly [37]. Regarding crystal structure, PEKK varies from PEEK since it is polymorphic and can crystallize in various crystalline unit cells depending on the crystallization technique. Since PEKK is a strong material, it can be used as a biomaterial for dental implants. Alsadon et al. have recently looked at how PEKK bilayer crowns wear over time and compared them to zirconia and nickel-chromium crowns.

In term of digital manufacturing, PEKK offers more printable polymers than PEEK due to the position of the ketone bonds in the aromatic ring being able to change, allowing for melting temperature and crystallization rate changes. It has a more pleasing aesthetic appearance and better wear and friction. In PEKK polymers, adding a second ketone group to the polymer backbone creates repeat units of two isomeric forms. As a result, it will be less affected by cooling, allowing for better adhesion to the tray and less warping. Regarding antibacterial activity, compared to PEEK, which is used in the orthopedic industry, PEKK exhibits less bacterial adherence on its surface. Without antibiotics, they an approximately 50% reduction in *Pseudomonas aeruginosa* adhesion and growth was observed on PEKK following five days of incubation. Other than that, the adherence to *Staphylococcus epidermidis* was 37% less on the surface of PEKK [21]. Multiple studies have shown the anti-inflammatory action of PEKK and fewer bacteria adhesion compared to conventional polymethylmethacrylate (PMMA) and polyetheretherketone (PEEK) [22,38].

## 5. Application of High-Performance Polymer (HPPs) in Dentistry

Aesthetics become a great concern for patients undergoing dental procedures. In order to improve patient satisfaction, aesthetic factors must be taken into account while designing dental procedures, particularly prosthetic treatments. Patients who, for aesthetic reasons, prefer RPDs with non-metal clasps using thermoplastic resin-based removable partial dentures (RPDs), face the drawback of metal elements lacking rigidity and inadequate support on the abutment teeth. In order to incorporate a metal framework into non-metal clasp dentures, the dimensional accuracy of the thermoplastic denture base resins should be equivalent or greater than the precision required for conventional acrylic resin. A previous study investigated the fitting accuracy of thermoplastic denture base resins used for non-metal clasp denture compared to a conventional acrylic resin. The results state that incorporating metal framework into thermoplastic RPD still suffers from a lack of support on abutment teeth, even though thermoplastic resin RPDs were originally developed in the 1950s for patients with allergies to acrylic resin [39]. This is because the thermoplastic resins, such as polymethylmethacrylate (PMMA), polycarbonate, and polyethylene terephthalate resin, have low elastic modulus for non-metal clasp in RPD. Hence, this review recommends HPP incorporation into thermoplastic to get a better result in terms of biological and mechanical properties based on the PEEK and PEKK.

However, in preliminary research and application, PEEK and PEKK are extensively utilized as dental implants, temporary abutments, obturators, clasps for dentures, and others due to their excellent biological, mechanical, cosmetic, and handling features. [40]. More than 1300 dental implant systems with various sizes, shapes, and surface properties are accessible on the dentistry market [41]. Over the past two decades, researchers and dentists have worked to perfect metal-free alternatives to traditional dental implants, abutments, and restorations. Zirconium dioxide is one such material [42,43]. Unfortunately, this material has low-temperature deterioration and a high Young’s modulus [44,45].

In depth studies of cutting-edge approaches for enhancing PEEK’s use in dental im-plants, prosthodontics, and orthodontics have been conducted. PEEK has been used in implant material, removable protheses and their components, and as a framework in fixed prostheses [10,17]. PEEK is a “isoelastic” implant material because it possesses an elastic modulus of 3–4 GPa and a Young modulus (18 GPa) comparable to human cortical bone [22]. Meanwhile, with a partial denture framework made of PEEK, patients are more comfortable due to its strength and digital design which prioritizes individual anatomy. Thus, PEEK frameworks represent an excellent shock absorbent during mastication while offering resistance to decay and abrasion. Since PEEK is chemically inert, numerous techniques have been utilized to establish a strong bond with veneering materials. PEEK can construct clasps and dentures by CAD CAM systems because of its lightweight, superior biological, aesthetic, and mechanical properties [41]. However, PEEK has less of a stress shielding effect on the surrounding bone than contemporary metal alloys [46,47,48,49,50].

However, PEEK has the disadvantage of an opaque or greyish color [51]. Because of its inferior coloration, it cannot be used to restore the front teeth for the sake of aesthetics [52,53]. PEEK’s high mechanical strength has led to its employment in a variety of applications, including as an implant material, CAD/CAM material, coating material, and abutment material [54]. A good feature of this material was that it could be mixed with other substances [55]. For restoration purposes, PEEK showed higher fracture resistance than all aesthetic post materials [56]. In order to prove the ability of fracture resistance of PEEK polymer, a comparative study was performed between polymer-infiltrated ceramic, fiber-reinforced composite post, and PEEK. Incorporating carbon fibers into PEEK composites led to a greater elastic modulus (18 GPa) (CFR-PEEK) [57], making them comparable to human cortical bone and dentin [58].

These techniques, however, pose additional hurdles. Given PEEK’s low surface energy and inertness, achieving sufficient binding strength to resin composites remains difficult [13,59,60]. The reinforcements of the PEEK polymer into 3D printed denture base resin were shown to improve the overall mechanical properties of the structure. Nevertheless, PEEK is less aesthetic and rarely has an optical appearance that can blend well with an intraoral profile. It is also inert to bonding and relatively expensive compared to the other high-performance polymer alternative PEKK group [61]. This article discussed the advances polymer (PEEK and PEKK) based biomaterials for dental applications, including novel properties and innovative engineering methods for the preliminary study of HPPs properties and surface modifications, which are significant in prosthetic dentistry.

## 6. Additive Manufacturing Application in Dentistry

Additive manufacturing (AM), or three-dimensional (3D) printing, refers to all those processes where layers of materials are added to build a complete three-dimensional product. The basic principle of the additive manufacturing approach is creating a three-dimensional through CAD model of the desired component and feeding it to an additive manufacturing (AM) machine in STL file format. For many industry insiders and hobbyists, the development of 3D printing has been rapid. It has been utilized for about 30 years in the medical, industrial, design, engineering, and manufacturing fields for rapid prototyping [62].

**Figure 5:**
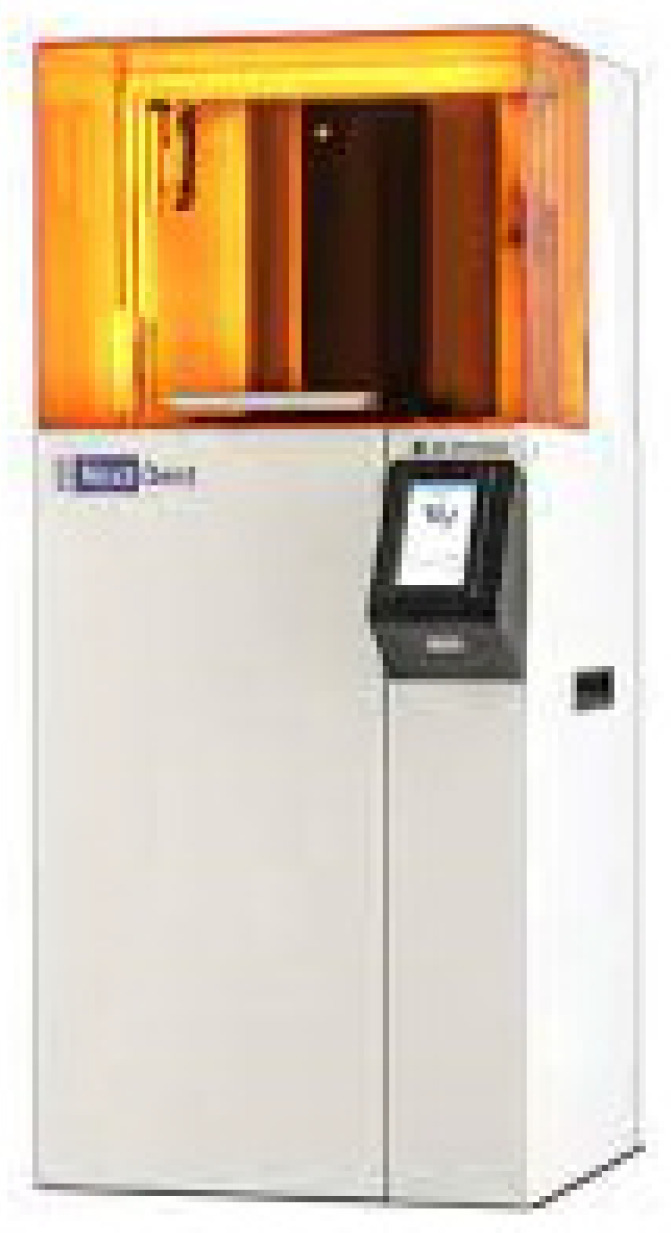
Next Dent 3D printing machine.

Digital dentistry, medical technology, particularly three-dimensional (3D) printing, is becoming the norm in the dental prosthesis industry, necessitating materials that exhibit a holistic feature in terms of printability, biocompatibility, degradability, and mechanical qualities. Additive manufacturing, or 3D printing (Figure 5), technologies have a variety of clinical applications in medicine and significant applications in dentistry. With the combination of advancements in 3D scanning technologies, 3D printing can immediately receive CAD data and instantly produce a new digital model for these traditional manufacturing processes [39].

Clinical applications of digital imaging and 3D printing in dentistry vary beyond prosthodontics and oral and maxillofacial surgery to oral implantology and orthodontics, endodontics, and periodontics [63]. The use of 3D printing in dentistry can streamline the complex workflow involved in the development of dental equipment, making the process more affordable and individualized for patients [61]. Due to its ability to apply a wide range of shapes directly related to the biological location, additive manufacturing is well-known for its application in dentistry. The umbrella term “light curing technology” encompasses all types of 3D printing that involve photosensitive resin components that are cured and shaped under light irradiation [64].

By leveraging 3D printing technology, dentures were fabricated more rapidly with fewer steps in the process, decreasing the likelihood of mistakes being made [58]. However, research on 3D printing’s potential to create complete dentures is still in infancy [38]. Tasaka et al. stated that photopolymerization spray helps make a more accurate base for a full denture than traditional thermal polymerization [65]. Yoon et al. [66] examined the tissue surface’s adaptability by contrasting the conventional packaging pressing technique (PAP) with a CAD/CAM complete denture base using either 5-axis milling or DLP generation.

Nonetheless, the current problems associated with 3D printed dentures are the inferiority of the structural integrity compared to conventional, and CAD-CAM derived denture fabrication techniques. The rapid development of digital technology will lead to an increase in new 3D printed (additive manufacturing) and CAD-CAM (subtractive manufacturing) materials for denture base fabrication. With the further development of digital technology, this additive manufactured denture will allow the fabrication of digital dentures in a quick turnaround. Consequently, fewer clinic visits are required, and problems associated with CAD-CAM technology such as material wastage, high initial equipment and production cost, maintenance of requirements and others, are eliminated. Furthermore, using standard processing methods for processing dentures necessitates a tedious human workforce on the part of the dental technician, which can increase the likelihood of human mistakes due to the step-by-step nature of the procedure, the essence of the clinical and laboratory processes involved in conventional fabrication.

Another limitation commonly associated with conventional denture fabrication, manufacturing, and processing is that multiple visits are required for the patients to attend dental practice. The traditional fabrication method of a denture involves a step-by-step clinical and laboratory intervention. The intervention requires the construction of specific equipment individualized for each patient, such as a specialized tray for impression, bite registration block, and multiple visits necessary to confirm the laboratory procedure with clinical findings.

Based on the fabrication of prostheses, subtractive machining technology is currently dominant. Still, it is unavoidable that additive processing routes of layered fabrication, such as FDM, SLA, and SLS, will emerge. In an additive system, preproduction patterns, e.g., made of wax or plastic, can be turned into definitive prostheses and workpieces directly in metals, resins, or ceramics. Regardless of the process, additive manufacturing is distinguished from subtractive manufacturing by the following characteristics: progressive vertical item construction, absence of material waste, manufacture of large objects, passive production involving no force application, and fabrication of precise details.

In prosthodontics, conventional fabrication techniques include taking an impression of the treated area, casting a stone model, and creating a wax pattern. The wax design is removed, and a more permanent substance, such as metal, ceramic, acrylic, or silicone, is invested. With added time and money, errors and mistakes in processing are possible outcomes. Modern digitalization in denture manufacturing protocols, including subtractive and addictive manufacturing assisted with computer-aided design and manufacturing (CAD-CAM), have been introduced to overcome the traditional method’s limitations and provide additional benefits, such as the advantage of facilitating the ideal fabrication of facial prostheses.

An alternate method for fabricating oral and facial prostheses has emerged owing to the development of computer-aided manufacturing and the medical use of this technological breakthrough in the industry. Because of developments in computer-designed technology, CAD/CAM has had a profound impact on the dental industry. Many branches of dentistry and oral and maxillofacial surgery, such as prosthodontics and orthodontics, are beginning to use digital medical therapeutic interventions. Classifying CAD/CAM production as either subtractive or additive is possible. Fabrication in subtractive manufacturing, including machining and milling, involves removing material from a solid block. Subtractive manufacturing is driven by computer numerical control (CNC).

Despite that, CAM is a software that automatically turns the CAD model into a tool path for the CNC machine. In dentistry, crowns, posts, inlays, and on-lays can be made with subtractive manufacturing. CNC tools use this computer-aided manufacturing (CAM) data in STL file format to make parts. However, milling and 3-D printing start from the same place, which the prosthesis is digitally designed with the CAD software. After that, the steps of making them are very different. In the subtractive method, a PMMA block and a five-axis milling machine that is controlled by a computer are used. If full dentures were made from PMMA blocks that had already been polymerized, there might not be any holes or shrinkage caused by the resin polymerizing. Thus, material properties would be enhanced, and residual monomer levels would be diminished [67]. Subsequently, the residual monomer content of the entire milled dentures would not be considerably lower than that of traditional heat-polymerized complete dentures and significantly lower than that of auto-polymerizing resin complete dentures [68].

Photopolymerization-based 3D printing processes, mainly digital light processing (DLP) and stereolithography (SLA), can create zirconia components [69]. From the viewpoint of manufacturing dental zirconia components, such as dental crowns, abutments, and implants, 3D printing techniques have recently drawn increased attention as an alternative to traditional dental computer-aided design and computer-aided manufacturing (CAD/CAM) technology [68]. The robust cutting tool and material losses from dental CAD/CAM can also be avoided using an additive manufacturing approach. Young’s paper mentioned that digital light processing (DLP) could construct zirconia components by photopolymerizing thin layers of a zirconia suspension in a layer-by-layer fashion according to predetermined 3D designs. Consequently, a high degree of design freedom and precision can be obtained, essential for producing dental zirconia ceramics with external and interior shapes customized for specific patients [70,71,72,73].

**Figure 6:**
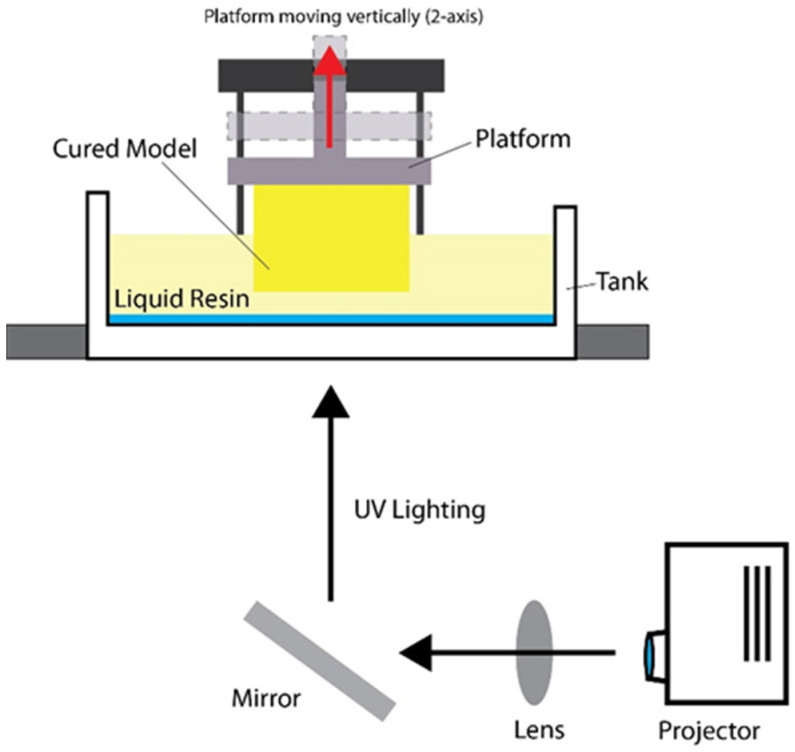
DLP printing mechanism uses projection.

Other than DLP, stereolithography (SLA) is one of the earliest 3D printing technologies. Stereolithography is widely used in the dental field to fabricate resin materials, such as surgical templates for oral and extraoral implant insertion and pre-prosthetic surgery. SLA works with liquids as photopolymer resin is applied and cured using a UV laser. The gadget also includes a model-building platform and an ultraviolet (UV) laser [74] to cure the photosensitive liquid resin. As a particular instance, stereolithography (SLA) would be executed layer by layer to create models and production parts. During the building process, an ultraviolet (UV) laser polymerizes the liquid resin while the construction platform is submerged in the resin. The built platform then moves an amount equal to the layer’s thickness, and the uncured resin is spread over the top of the preceding layer of the same mechanism to the DLP mechanism used for the projector (Figure 6) [75].

There are two distinct ways to move the platform within the SLA technology. The first motion of the platform is a downward motion from above. The building platform soaked in the resin reservoir is covered with a layer of resin to create a layer of resin. Following the completion of the laser inspection of the initial layer, the building platform travels downward, and a wheel adjacent to it adds a further layer of resin. Iterations of the build cycle are carried out till the object is produced [31].

Other than DLP and SLA, selective laser sintering (SLS) has become an emerging digital dental application. SLA relies on a laser as its power source to sinter powdered material, fusing it to form a solid. However, the choice of materials is a crucial distinction between SLA and SLS. SLA cures resin while SLS sinters the powder of the materials. However, SLS is only working with polymers not metal. Nonetheless, only a few studies support their use in clinical settings, as new fabrication procedures have been developed for removable partial denture (RPD) frameworks. Studies validating the clinical application of these techniques are scarce. Based on the previous study by Samuel in 2022, RPD frameworks made with the SLS technique presented adaptation with higher variability than cast RPD [74].

PEKK has heralded the advent of emerging polymer materials with improved biocompatibility, durability, and flexibility, as well as those that are more visually attractive and cost-effective, representing a significant advancement for detachable prosthetics. While CAD-CAM technology has proven to be superior in the production of dentures, the subtractive approach used by milling machines introduces more waste, making it less environmentally acceptable. In terms of environmental impact, energy consumption, production cost, and other criteria, 3D printing using additive manufacturing models is more advantageous. However, the manufacture of digital oral prostheses by 3D printing is still in its early stages, with frequent issues, such as inadequate overall structural integrity, color instability, and easy adherence to undesirable oral microbiomes [76].

## 7. Utilization of Ultra-Height Molecular Weight Polyethylene (UHMWPE) and High-Performance Polymer (HPPs) as Dental Materials

PEEK and UHMWPE are types of thermoplastics, but UHMWPE is a type of polyethylene plastic that is commonly used in robotics. It has a high level of flexibility and is lightweight compared to other thermoplastics. UHMWPE is a versatile material that can be used in various areas of dentistry, such as prosthodontics, restorative dentistry, endodontics, paedodontics, orthodontics, and periodontics. Table 1 shows a comparison properties of UHMWPE, PEEK, and PEKK as dental materials [77,78], indicating that PEKK is an advantageous and emerging biomaterial for dental applications.

The utilization of UHMWPE is varied in dentistry. In orthodontics, UHMWPE ribbon-reinforced composites are used as lingual retainers to solve issues related to traditional stainless steel wire retainers. Polyethylene fiber-reinforced composites are used to splint teeth that have become mobile due to periodontal lesions following trauma. Recently, UHMWPE fiber-reinforcement systems have been widely used as endodontic posts to conserve remaining sound dental structures and reduce the risk of root canal perforation. UHMWPE fibers placed in the occlusal third of restorations have shown even higher fracture resistance [79]. Additionally, UHMWPE ribbons have been used with composite resin to restore single anterior edentulous spaces in prosthodontic treatment. In addition to prosthodontics treatment, UHMWPE ribbons have been used to restore single anterior edentulous space using Ribbond ribbon and composite resin [79].

Dentistry has been evolving to incorporate high-performance polymers (HPP) as advanced materials due to their enhancing properties and potentially lower rehabilitation costs. PAEK is a semicrystalline thermoplastic polymer with excellent mechanical and chemical resistance [80], as well as great machinability [81]. Its structure affects its properties, with more ketone groups leading to more polarity and rigidity, resulting in a higher glass transition temperature and melting point. PEEK and PEKK have physical and mechanical characteristics similar to bone and natural tooth structure, making them suitable substitutes for metals and other materials due to their non-allergic properties and acceptable aesthetics. This review provides insight into the properties and applications of polymers, particularly PEEK and PEKK, in dental applications, including prosthetics, implant frameworks, abutments, crowns, and orthodontic wires, as well as restorative dentistry, and demonstrates their potential for clinical uses.

In recent years, a novel class of similar materials has been created from the generic family of polyaryletherketones (PAEK). Because of its biocompatibility and advantageous physical and mechanical qualities, the material polyetherether-ketone (PEEK), a semicrystallize thermosoftening polymer, has the potential to serve as a framework for fixed and detachable prostheses [78]. PEEK has been introduced as metal-free material in implant, which enhancing the properties of biomechanical. PEEK has flexural strength of 140–170 MPa [57] and elastic modulus of 18 GPa after being reinforced with carbon fiber. In comparison with elastic modulus of titanium that is 110 GPa, the elastic modulus of PEEK is close to the elastic modulus of the bone [11,12]. Due to mastication or any parafunctional habits such as bruxism, titanium abutment or screws are replaced by PEEK as it does not cause hypersensitivity unlike titanium and can bear forces such as titanium [47]. Other than that, PEEK is used as a prosthetic material in removable partial dentures (RPDs), fixed partial dentures (FPDs), and crowns. In removable prosthesis, it can be used as clasps and can replace cobalt-chromium alloys [17]. PEKK shows superior shock absorbance and less stress concentration of 49 MPa, whereas PEEK shows higher stress on the base of prosthesis of 58 MPa [78]. The lesser the stress concentration, the lesser the fracture risk on the denture base. For irrigation during root canal treatment, tips used to irrigate the canals can be made from PEEK polymer as it is chemical resistant and does not react with irritants, such as sodium hypochlorite, chlorhexidine, and ethylenediaminetetraacetic acid [12,82]. In terms of orthodontics, ceramic brackets have some shortcomings in relation to the force applied by brackets being comparatively less, which in turn increases the treatment span. To overcome this limitation, PEEK is a more feasible material that can be used as an alternative to conventional self-cure and heat cure space maintainers, making it favorable and aesthetically pleasing for the patient when combined with digital technology [83].

PEKK is a type of PAEK polymer that has excellent physical, mechanical, and biological properties. It is a viable alternative to titanium in implants because it is more compatible with other materials. When combined with titanium, PEKK provides long-lasting retention [12]. PEKK clasps can also be used in removable prostheses and as an alternative to nylon inserts because they are more retentive and abrasive [84]. With the use of additive manufacturing, PEKK polymer can create highly retentive and perfectly fitted dentures with surface modifications. Additionally, PEKK can be used as endodontic posts and endo crowns [85,86]. Custom-made PEKK posts have been shown to have stronger bonds than prefabricated ones. In orthodontic treatment, PEKK materials can replace metal ortho wires and provide the necessary force for proper alignment.

## 8. Conclusions

Dental prostheses are crucial in preserving older people’s facial features and attractiveness. In recent years, patients have favored a metal-free, tooth-colored, and lightweight prosthesis. The passive nature of additive techniques enables the manufacturing of more complex build structures without excessive force and substantially less non-recyclable waste than subtractive techniques. Since there are so many available additive techniques and materials, dentistry might need them in various ways. PEEK was used to make the framework of fixed denture prostheses. It can also prepare a dental crown with a veneering composite coating on the front. Therefore, many ways have been shown to make it easier to bond the PEEK with the resin composite crown. PEKK is also made without metal, so it can be used as an alternative to titanium implants. PEKK abutments are adjustable and can be used with different veneering materials. It can also provide the structural support for a prosthesis held in place by implants. Combining the PEKK attachment system with titanium could be a way to make an implant prosthesis that stays in place for a long time. However, additive manufacturing is another way to make things, even though it has yet to be used much in dentistry because of its high price.

## Figures and Tables

**Table 1:** Comparison properties of different materials.

Properties	PEKK	PEEK	UHMWPE
Specific Gravity	1.27 g/cm^3^	1.30 g/cm^3^	0.933 g/cm^3^
Glass Transition Temperature	162 °C	143 °C	−110 °C
Melting Point	305 °C	340 °C	200–220 °C
Tensile Strength	105 MPa	90–100 MPa	48 MPa
Young Modulus	5.10 GPa	3.70 GPa	0.69 GPa
Operating temperature	300 °C	250 °C	<100 °C

## References

[1] Alla R., Raghavendra K.N., Vyas R., Konakanchi A. (2015). Conventional and contemporary polymers for the fabrication of denture prosthesis: Part I–overview, composition and properties. Int. J. Appl. Dent. Sci..

[2] Rueggeberg F.A. (2002). From vulcanite to vinyl, a history of resins in restorative dentistry. J. Prosthet. Dent..

[3] Ramsay S.E., Whincup P.H., Watt R.G., Tsakos G., Papacosta A.O., Lennon L.T., Wannamethee S.G. (2015). Burden of poor oral health in older age: Findings from a population-based study of older British men. BMJ Open.

[4] Uriciuc W.A., Vermesan H., Tiuc A.E., Ilea A., Bosca A.B., Popa C.O. (2021). Casting over metal method used in manufacturing hybrid cobalt-chromium dental prosthetic frameworks assembles. Materials.

[5] World Health Organization (1980). International Classification of Impairments, Disabilities, and Handicaps: A Manual of Classification Relating to the Consequences of Disease.

[6] Kidd E.A., Giedrys-Leeper E., Simons D. (2000). Take two dentists: A tale of root caries. Dent. Update.

[7] Wiesli M.G., Özcan M. (2015). High-performance polymers and their potential application as medical and oral implant materials: A review. Implant. Dent..

[8] Stawarczyk B., Wimmer T., Jahn D., Sener B., Roos M., Schmidlin P.R. (2013). Polyetheretherketone(PEEK)—A suitable material for fixed dental prostheses?. J. Biomed. Mater. Res. B Appl. Biomater..

[9] Jamari J., Ammarullah M.I., Santoso G., Sugiharto S., Supriyono T., Prakoso A.T., Basri H., van der Heide E. (2022). Computational Contact Pressure Prediction of CoCrMo, SS 316L and Ti6Al4V Femoral Head against UHMWPE Acetabular Cup under Gait Cycle. J. Funct. Biomater..

[10] Fuhrmann G.S.M., Freitag-Wolf S., Kern M. (2014). Resin bonding to three types of polyaryletherketones (PAEKs)-durability and influence of surface conditioning. Dent. Mater..

[11] Moon S.M., Ingalhalikar A., Highsmith J.M., Vaccaro A.R. (2009). Biomechanical rigidity of an all-polyetheretherketone anterior thoracolumbar spinal reconstruction construct: An in vitro corpectomy model. Spine J..

[12] Xin H., Shepherd D., Dearn K. (2013). Strength of poly-etherether-ketone: Effects of sterilization and thermal ageing. Polym. Test..

[13] Ates S.M., Caglar I., Duymus Z. (2018). The effect of different surface pretreatments on the bond strength of veneering resin to polyetheretherketone. J. Adhes. Sci. Technol..

[14] Zhou L.Q.Y., Zhu Y., Liu H., Gan K., Guo J. (2014). The effect of different surface treatments on the bond strength of PEEK composite materials. Dent. Mater..

[15] Koutouzis T.R.J., Lundgren T. (2011). Comparative soft and hard tissue responses to titanium and polymer healing abutments. J. Oral Implantol..

[16] Najeeb S.Z.M., Khurshid Z., Siddiqui F. (2016). Applications of polyetheretherketone (PEEK) in oral implantology and prosthodontics. J. Prosthodont. Res..

[17] Tannous F., Steiner M., Shahin R., Kern M. (2012). Retentive forces and fatigue resistance of thermoplastic resin clasps. Dent. Mater..

[18] Rosentritt M.P.V., Behr M., Sereno N., Kolbeck C. (2015). Shear bond strength between veneering composite and PEEK after different surface modifications. Clin. Oral Investig..

[19] Kurtz S.M., Devine J.N. (2007). PEEK biomaterials in trauma, orthopedic, and spinal implants. Biomaterials.

[20] Elawadly T., Radi I.A.W., El Khadem A., Osman R.B. (2017). Can PEEK Be an Implant Material? Evaluation of Surface Topography and Wettability of Filled Versus Unfilled PEEK with Different Surface Roughness. J. Oral Implantol..

[21] Skinner H.B. (1988). Composite technology for total hip arthroplasty. Clin. Orthop. Relat. Res..

[22] Alqurashi H., Khurshid Z., Syed A.U.Y., Habib S.R., Rokaya D., Zafar M.S. (2021). Polyetherketoneketone (PEKK): An emerging biomaterial for oral implants and dental prostheses. J. Adv. Res..

[23] Song C.H., Choi J.W., Jeon Y.C., Jeong C.M., Lee S.H., Kang E.S., Yun M.J., Huh J.B. (2018). Comparison of the microtensile bond strength of a polyetherketoneketone (PEKK) tooth post cemented with various surface treatments and various resin cements. Materials.

[24] Swier S., Gasa J., Shaw M.T., Weiss R.A. (2004). Sulfonation Reaction Kinetics of Poly(Ether Ketone Ketone) (PEKK) Using a Mixture of Concentrated and Fuming Sulfuric Acid. Ind. Eng. Chem. Res..

[25] Hussain M., Naqvi R.A., Abbas N., Khan S.M., Nawaz S., Hussain A., Zahra N., Khalid M.W. (2020). Ultra-High-Molecular-Weight-Polyethylene (UHMWPE) as a Promising Polymer Material for Biomedical Applications: A Concise Review. Polymers.

[26] Falkensammer F., Arnetzl G.V., Wildburger A., Freudenthaler J. (2013). Color stability of different composite resin materials. J. Prosthet. Dent..

[27] Sinha N., Gupta N., Reddy K.M., Shastry Y. (2017). Versatility of PEEK as a fixed partial denture framework. J. Indian Prosthodont. Soc..

[28] Kessler A., Hickel R., Reymus M. (2020). 3D printing in dentistry—State of the art. Oper. Dent..

[29] Perez-Martin H., Mackenzie P., Baidak A., Brádaigh C.M.Ó., Ray D. (2021). Crystallinity studies of PEKK and carbon fibre/PEKK composites: A review. Compos. Part B.

[30] Qin L., Yao S., Zhao J., Zhou C., Oates T.W., Weir M.D., Wu J., Xu H.H. (2021). Review on development and dental applications of polyetheretherketone-based biomaterials and restorations. Materials.

[31] Kewekordes T., Wille S., Kern M. (2018). Wear of polyetherketoneketones—Influence of titanium dioxide content and antagonistic material. Dent. Mater..

[32] Guo R., McGrath J., Matyjaszewski K., Möller M. (2012). Polymer Science: A Comprehensive Reference.

[33] Stawarczyk B., Jordan P., Schmidlin P.R., Roos M., Eichberger M., Gernet W., Keul C. (2014). PEEK surface treatment effects on tensile bond strength to veneering resins. J. Prosthet. Dent..

[34] Vos T., Flaxman A.D., Naghavi M., Lozano R., Michaud C., Ezzati M., Shibuya K., Salomon J.A., Abdalla S., Aboyans V. (2012). Years lived with disability (YLDs) for 1160 sequelae of 289 diseases and injuries 1990–2010, A systematic analysis for the Global Burden of Disease Study 2010. Lancet.

[35] Yuan B., Cheng Q., Zhao R., Zhu X., Yang X., Yang X., Zhang K., Song Y., Zhang X. (2018). Comparison of osteointegration property between PEKK and PEEK: Effects of surface structure and chemistry. Biomaterials.

[36] Olivares-Navarrete R., Hyzy S.L., Gittens R.A., Schneider J.M., Haithcock D.A., Ullrich P.F., Slosar P.J., Schwartz Z., Boyan B.D. (2013). Rough titanium alloys regulate osteoblast production of angiogenic factors. Spine J..

[37] Moore R., Beredjiklian P., Rhoad R., Theiss S., Cuckler J., Ducheyne P., Baker D.G. (1997). A comparison of the inflammatory potential of particulates derived from two composite materials. J. Biomed. Mater. Res. Off. J. Soc. Biomater. Jpn. Soc. Biomater..

[38] Wada J., Fueki K., Yatabe M., Takahashi H., Wakabayashi N. (2014). A comparison of the fitting accuracy of thermoplastic denture base resins used in non-metal clasp dentures to a conventional heat-cured acrylic resin. Acta Odontol. Scand..

[39] Tian Y., Chen C., Xu X., Wang J., Hou X., Li K., Lu X., Shi H., Lee E.S., Jiang H.B. (2021). A review of 3D printing in dentistry: Technologies, affecting factors, and applications. Scanning.

[40] Lesmes D., Laster Z. (2011). Innovations in dental implant design for current therapy. Oral Maxillofac. Surg. Clin. N. Am..

[41] Nakamura K., Kanno T., Milleding P., Örtengren U. (2010). Zirconia as a dental implant abutment material: A systematic review. Int. J. Prosthodont..

[42] Özkurt Z., Kazazoğlu E. (2011). Zirconia dental implants: A literature review. J. Oral Implantol..

[43] Akagi K., Okamoto Y., Matsuura T., Horibe T. (1992). Properties of test metal ceramic titanium alloys. J. Prosthet. Dent..

[44] Kelly J.R., Denry I. (2008). Stabilized zirconia as a structural ceramic: An overview. Dent. Mater..

[45] Ma R., Tang T. (2014). Current Strategies to Improve the Bioactivity of PEEK. Int. J. Mol. Sci..

[46] Wang H., Xu M., Zhang W., Kwok D.T., Jiang J., Wu Z., Chu P.K. (2010). Mechanical and biological characteristics of diamond-like carbon coated poly aryl-ether-ether-ketone. Biomaterials.

[47] Mishra S., Chowdhary R. (2019). PEEK materials as an alternative to titanium in dental implants: A systematic review. Clin. Implant. Dent. Relat. Res..

[48] Park P.J., Lehman R.A. (2020). Optimizing the Spinal Interbody Implant: Current Advances in Material Modification and SurfaceTreatment Technologies. Curr. Rev. Musculoskelet. Med..

[49] Deng Y., Zhou P., Liu X., Wang L., Xiong X., Tang Z., Wei J., Wei S. (2015). Preparation, characterization, cellular response and invivo osseointegration of polyetheretherketone/nano-hydroxyapatite/carbon fiber ternary biocomposite. Colloids Surf. B Biointerfaces.

[50] Wachtel A., Zimmermann T., Sutel M., Adali U., Abou-Emara M., Muller W.D., Muhlemann S., Schwitalla A.D. (2019). Bacterialleakage and bending moments of screw-retained, composite-veneered PEEK implant crowns. J. Mech. Behav. Biomed. Mater..

[51] Khalesi R., Abbasi M., Shahidi Z., Tabatabaei M.H., Moradi Z. (2020). Interfacial Fracture Toughness Comparison of Three IndirectResin Composites to Dentin and Polyether Ether Ketone Polymer. Eur. J. Dent..

[52] Bathala L., Majeti V., Rachuri N., Singh N., Gedela S. (2019). The Role of Polyether Ether Ketone (Peek) in Dentistry—A Review. J. Med. Life.

[53] Kaleli N., Sarac D., Kulunk S., Ozturk O. (2018). Effect of different restorative crown and customized abutment materials on stressdistribution in single implants and peripheral bone: A three-dimensional finite element analysis study. J. Prosthet. Dent..

[54] Gan K., Liu H., Jiang L., Liu X., Song X., Niu D., Chen T., Liu C. (2016). Bioactivity and antibacterial effect of nitrogen plasmaimmersion ion implantation on polyetheretherketone. Dent. Mater..

[55] Knaus J., Schaffarczyk D., Colfen H. (2020). On the Future Design of Bio-Inspired Polyetheretherketone Dental Implants. Macromol. Biosci..

[56] Haralur S.B. (2021). Fracture resistance of endodontically treated teeth restored with various esthetic posts. Technol. Health Care.

[57] Schwitalla A., Müller W.-D. (2013). PEEK Dental Implants: A Review of the Literature. J. Oral Implantol..

[58] Wang W., Luo C.J., Huang J., Edirisinghe M. (2019). PEEK surface modification by fast ambient-temperature sulfonation for bone-implant applications. J. R. Soc. Interface.

[59] Stawarczyk B., Keul C., Beuer F., Roos M., Schmidlin P.R. (2013). Tensile bond strength of veneering resins to PEEK: Impact of different adhesives. Dent. Mater. J..

[60] Panayotov I.V., Orti V., Cuisinier F., Yachouh J. (2016). Polyetheretherketone (PEEK) for medical applications. J. Mater. Sci. Mater. Med..

[61] Khng K.Y.K., Ettinger R.L., Armstrong S.R., Lindquist T., Gratton D.G., Qian F. (2016). In vitro evaluation of the marginal integrity of CAD/CAM interim crowns. J. Prosthet. Dent..

[62] Lin L., Fang Y., Liao Y., Chen G., Gao C., Zhu P. (2019). 3D printing and digital processing techniques in dentistry: A review of literature. Adv. Eng. Mater..

[63] Prechtel A., Reymus M., Edelhoff D., Hickel R., Stawarczyk B. (2020). Comparison of various 3D printed and milled PAEK materials: Effect of printing direction and artificial aging on Martens parameters. Dent. Mater..

[64] Layani M., Wang X., Magdassi S. (2018). Novel materials for 3D printing by photopolymerization. Adv. Mater..

[65] Tasaka A., Matsunaga S., Odaka K., Ishizaki K., Ueda T., Abe S., Yoshinari M., Yamashita S., Sakurai K. (2019). Accuracy and retention of denture base fabricated by heat curing and additive manufacturing. J. Prosthodont. Res..

[66] Yoon H.I., Hwang H.J., Ohkubo C., Han J.S., Park E.J. (2018). Evaluation of the trueness and tissue surface adaptation of CAD-CAM mandibular denture bases manufactured using digital light processing. J. Prosthet. Dent..

[67] Masri G., Mortada R., Ounsi H., Alharbi N., Boulos P., Salameh Z. (2020). Adaptation of complete denture base fabricated by conventional, milling, and 3-d printing techniques: An in vitro study. J. Contemp. Dent. Pract..

[68] Steinmassl P.A., Wiedemair V., Huck C., Klaunzer F., Steinmassl O., Grunert I., Dumfahrt H. (2017). Do CAD/CAM dentures really release less monomer than conventional dentures?. Clin. Oral Investig..

[69] Yang S.-Y., Koh Y.-H., Kim H.-E. (2023). Digital Light Processing of Zirconia Suspensions Containing Photocurable Monomer/Camphor Vehicle for Dental Applications. Materials.

[70] Galante R., Figueiredo-Pina C.G., Serro A.P. (2019). Additive manufacturing of ceramics for dental applications: A review. Dent. Mater..

[71] Khanlar L.N., Rios A.S., Tahmaseb A., Zandinejad A. (2021). Additive manufacturing of zirconia ceramic and its application in clinical dentistry: A review. Dent. J..

[72] Zakeri S., Vippola M., Levänen E. (2020). A comprehensive review of the photopolymerization of ceramic resins used in stereolithography. Addit. Manuf..

[73] De Camargo I.L., Morais M.N., Fortulan C.A., Branciforti M.C. (2021). A review on the rheological behavior and formulations of ceramic suspensions for vat photopolymerization. Ceram. Inter..

[74] Barazanchi A., Li K.C., Al-Amleh B., Lyons K., Waddell J.N. (2017). Additive technology: Update on current materials and applications in dentistry. J. Prosthodont..

[75] Pelletier S., Pelletier A., Al Dika G. Adaptation of removable partial denture rest seats in prostheses made with selective laser sintering or casting techniques: A randomized clinical trial. J. Prosthet. Dent..

[76] Ramburrun P., Pringle N.A., Dube A., Adam R.Z., D’Souza S., Aucamp M. (2021). Recent advances in the development of antimicrobial and antifouling biocompatible materials for dental applications. Materials.

[77] Selvam S., Marimuthu K. (2016). Development and Investigation of Mechanical Properties of PEEK Fine Particles Reinforced UHMWPE Composites. Int. J. Appl. Eng. Res..

[78] Villefort R.F., Diamantino P.J.S., Von Zeidler S.L.V., Borges A.L.S., Saavedra G.D.F.A., Tribst J.P.M. (2022). Mechanical response of PEKK and PEEK as frameworks for implant-supported full-arch fixed dental prosthesis: 3D finite element analysis. Eur. J. Dent..

[79] Agrawal M. (2014). Applications of ultrahigh molecular weight polyethylene fibres in dentistry: A review article. J. Adv. Med. Dent. Sci. Res..

[80] Choupin T. (2017). Mechanical Performances of PEKK Thermoplastic Composites Linked to Their Processing Parameters. Ph.D. Thesis.

[81] Aldhuwayhi S., Alauddin M.S., Martin N. (2022). The Structural Integrity and Fracture Behaviour of Teeth Restored with PEEK and Lithium-Disilicate Glass Ceramic Crowns. Polymers.

[82] Kucher M., Dannemann M., Modler N., Hannig C., Weber M.T. (2019). Effects of endodontic irrigants on material and surface properties of biocompatible thermoplastics. Dent. J..

[83] Guo H., Wang Y., Zhao Y., Liu H. (2020). BMC Computer-aided design of polyetheretherketone for application to removable pediatric space maintainers. Oral Health.

[84] Choi J.W., Yun B.H., Jeong C.M., Huh J.B. (2018). Retentive properties of two stud attachments with polyetherketoneketone or nylon insert in mandibular implant overdentures. Int. J. Oral Maxillofac. Implant..

[85] Lee K.S., Shin J.H., Kim J.E., Kim J.H., Lee W.C., Shin S.W., Lee J.Y. (2017). Biomechanical evaluation of a tooth restored with high performance polymer PEKK post-core system: A 3D finite element analysis. BioMed Res. Int..

[86] Güven M.Ç., Dayan S.Ç., Yıldırım G., Mumcu E. (2020). Custom and prefabricated polyetherketoneketone (PEKK) post-core systems bond strength: Scanning electron microscopy evaluation. Microsc. Res. Tech..

